# Supplementary material for: The Systems Biology Research Tool: evolvable open-source software
Source: BMC Syst Biol. 2008 Jun 29;2:55. doi: 10.1186/1752-0509-2-55 (PMC2446383; doi:10.1186/1752-0509-2-55)
Supplement: Additional file 1 — SBRT Archive. An archive of the current version of the Systems Biology Research Tool. [file 1752-0509-2-55-S1.zip › sbrt-1.4.0/doc/developers_guide/api/sbrt/kernel/math/algebra/class-use/LinearSystemSolver.html]

Uses of Interface sbrt.kernel.math.algebra.LinearSystemSolver


|  |  |  |
| --- | --- | --- |
| **Overview** \| **Package** \| **Class** \| **Use** \| **Tree** \| **Deprecated** \| **Index** \| **Help** | |  |
| PREV   NEXT | **FRAMES**    **NO FRAMES**     **All Classes** |  |


---


## **Uses of Interface sbrt.kernel.math.algebra.LinearSystemSolver**

| Packages that use LinearSystemSolver | |
| --- | --- |
| **sbrt.ext.mma.algebra** | Provides classes that allow communication with Mathematica for the purposes of algebra. |
| **sbrt.kernel.fba.comp** | Provides classes and interfaces for performing Flux Balance Analysis computations. |
| **sbrt.kernel.fba.proc** | Provides classes and interfaces for executing Flux Balance Analysis processes. |
| **sbrt.kernel.math.algebra.proc** | Provides classes and interfaces for executing algebra-related processes. |

Uses of LinearSystemSolver in sbrt.ext.mma.algebra

| Classes in sbrt.ext.mma.algebra that implement LinearSystemSolver | |
| --- | --- |
| `class` | `MathematicaSolver<V>`             This class is used to solve systems of linear equations using Mathematica. |

Uses of LinearSystemSolver in sbrt.kernel.fba.comp

| Methods in sbrt.kernel.fba.comp with parameters of type LinearSystemSolver | |
| --- | --- |
| `static LinearSystemSolution<java.lang.String>` | `FbaSolver.solve(Fluxome fluxome, LinearSystemSolver<java.lang.String> solver)`             Solves *Sv = 0*, where *S* is the stoichiometry matrix of the provided fluxome and *v* is the vector of fluxes.** |

Uses of LinearSystemSolver in sbrt.kernel.fba.proc

| Methods in sbrt.kernel.fba.proc with parameters of type LinearSystemSolver | |
| --- | --- |
| `void` | `FbaSolverProcess.setSolver(LinearSystemSolver<java.lang.String> solver)`             Sets the linear system solver to be used. |

Uses of LinearSystemSolver in sbrt.kernel.math.algebra.proc

| Methods in sbrt.kernel.math.algebra.proc with parameters of type LinearSystemSolver | |
| --- | --- |
| `void` | `LinearSolverProcess.setSolver(LinearSystemSolver<V> solver)`             Sets the linear system solver. |

---


|  |  |  |
| --- | --- | --- |
| **Overview** \| **Package** \| **Class** \| **Use** \| **Tree** \| **Deprecated** \| **Index** \| **Help** | |  |
| PREV   NEXT | **FRAMES**    **NO FRAMES**     **All Classes** |  |


---
